# Supplementary material for: Determinants of pregnancy-induced hypertension on maternal and foetal outcomes in Hossana town administration, Hadiya zone, Southern Ethiopia: Unmatched case-control study
Source: PLoS One. 2021 May 12;16(5):e0250548. doi: 10.1371/journal.pone.0250548 (PMC8115896; doi:10.1371/journal.pone.0250548)
Supplement: S1 File — (DOCX) [file pone.0250548.s001.docx]

**Pre tested English version questionnaire**

Title: ***Determinants of pregnancy induced hypertension on maternal and foetal outcomes in Hossana town administration, Hadiya zone, Southern Ethiopia: Unmatched case-control stud.***

Address: Phone (mob.) 0923406980/0913791113

Email: [gossabo2004@gmail.com](mailto:gossabo2004@gmail.com)

Name of the organization Wachemo University School of medicine & health science, department of nursing

**Information to study participants**

Greeting --------------------------------------------------------

My name is------------------------- I am instructor in wachemo University. I am planning to conduct my study on maternal, perinatal outcomes of pregnancy indiced hypertension and associated factor among pregnant mothers at Hossana town, in Hadiya Zone, Southern Ethiopia. I am going to ask you some questions that are not difficult to answer which help us Pilot for further study.

**Conformation of Consent to participate**

Do you understand all I have just told you and do you agree to participate in this study? If you agree to participate in this study, you will need to give consent.

Yes ----------------------continue No------------------------stop

**PARTICIPANT AGREEMENT:- Participant**: I have read the study information and understand its objective / have been briefly informed about the study that has been read to me. I have been asked if I have any questions, and these have been answered to my satisfaction. I freely agree to participate.

Agree disagree

**Data collector**: I certify that the nature and purpose, the potential benefits, and possible risks associated with participating in this research have been explained to the above individual, and the individual has consented/agreed to participate.

| Code | Variable | | | Category /response | Skip |
| --- | --- | --- | --- | --- | --- |
|  | **Part I: Socio-demographic variables** | | | |  |
| 101 | Age of women during the current pregnancy | | | ------- |  |
| 102 | Age at marriage | | | ------ |  |
| 103 | Educational status | | | 1. No formal education 2. Primary to high school 3. College & above |  |
| 104 | Occupation | | | 1. Household wife 2. Gov’t employee 3. NGO employee 4. Student |  |
| 105 | Marital status | | | 1. single 2. married 3. divorced 4. widowed |  |
|  | **Part II: Obstetric d and gynaecological history** | | | |  |
| 201 | Gravidity | | | 1. Primgravida 2. II 3. III & above |  |
| 202 | Parity | | | 1. 0 2. 1-2 3. 3-4 4. > 5 |  |
| 203 | Do you remember your LNMP of current conception? | | | 1. Yes 2. No | Skip to Q 205 |
| 204 | If your answer for Q203 is yes, what is GA in weeks ( on the occasion of current visit) | | | ---- |  |
| 205 | Did you attend ANC follow up? | | | 1. Yes 2. No | Skip to Q 207 |
| 206 | If your answer for Q 206 is yes, How many ANC follow-up do you have | | | 1. 1 2. 2 3. 3 4. 4 and above |  |
| 207 | Pregnancy | | | 1. Single 2. Multiple |  |
| 208 | Do you have any previously diagnosed chronic disease | | | 1. Yes 2. No | Skip to Q 211 |
| 209 | If your answer for Q 208 is yes Which diagnosed medical disorder do you have | | | List   1. ------ 2. ------ 3. ------- |  |
| 210 | Previous history of pregnancy Induced hypertension | | | 1. Yes 2. No |  |
|  | **Part III: Mothers status on admission** | | | |  |
| 301 | Chief complaint during admission | | | 1. Headache 2. Blurred vision 3. Epigastric pain 4. Nausea and vomiting 5. Convulsion 6. Edema (pedal) 7. Dizziness |  |
| 302 | Blood pressure during admission | | | ---- |  |
| 303 | Condition of patient presentation on arrival to Hospital | | | 1. Conscious 2. Semi -conscious 3. comatose 4. convulsion |  |
| 304 | Does mother developed PIH (based on the assessment and documents ) | | | 1. yes 2. No |  |
| 305 | Category of hypertension during admission | | | 1. chronic hypertension 2. preeclampsia 3. Eclampsia 4. Peeclampsia superimposed on chronic HTN |  |
|  | **Part IV: Investigation done** | | | |  |
| 401 | Does blood group & Rh done | | 1. Yes 2. No | |  |
| 402 | Blood group and Rh type | | -------, ---- | |  |
| 403 | Protein urea | | 1. +1 2. +2 3. +3 4. > +4 5. Note done | |  |
| 404 | Platelet count | | 1. <100,000 2. 100,000 – 150,000 3. >150,000 4. Not done | |  |
| 405 | Does LFT done | | 1. Yes 2. No | | Skip to Q 407 |
| 406 | Result | | AST------  ALT -----  ALP ----- | |  |
| 407 | Does RFT done | | 1. Yes 2. No | | Skip to Q 501 |
| 408 | Result | | Creatinen ------  BUN ------- | |  |
|  | **Part V: onset of labor and delivery** | | | |  |
| 501 | Which type of onset of labor she had | 1. Spontaneous 2. Induction | | |  |
| 502 | Length of admission /day | ------- | | |  |
| 503 | Mode of the delivery | 1. Spontaneous vaginal delivery 2. Cesareans section | | |  |
| 504 | If answer for Q 503 is 2, what was the indication | 1. Uncontrolled peeclampsia 2. Happening of Eclampsia 3. Failure to Induction or augmentation 4. Abroptio placenta 5. Fetal distress 6. Others specify ---- | | |  |
|  | **Part VI: maternal and foetal outcomes** | | | |  |
| 601 | Is there any maternal complication | 1. Yes 2. No | | |  |
| 602 | If answer for Q 601 is yes, Which one | 1. HELLP Syndrome 2. Eclampsia 3. DIC 4. Cardiac failure 5. Renal failure 6. PPH 7. Pulmonary edema 8. Others specify | | |  |
| 603 | Maternal death | 1. Yes 2. No | | |  |
| 604 | Fetal outcome of delivery | 1. A live birth 2. Alive but admitted in NICU 3. Still birth | | |  |
| 605 | IF life birth, what was APGAR score for Neonate A | 1. Within 1^st^ minute ----- 2. At 5 minute ----- | | |  |
| 606 | What was fetal weight per Kg | 1. < 1.5 2. 1.5- 2.5 3. 2.5-4.0 4. > 4.0 | | |  |
| 607 | Gestational age at delivery | 1. Pre-Term 2. Term 3. Post term | | |  |
| 608 | Neonatal death | 1. Yes 2. No | | |  |
| 609 | Does a neonate had IUGR | 1. yes 2. No | | |  |
