# Supplementary material for: Determinants of pregnancy-induced hypertension on maternal and foetal outcomes in Hossana town administration, Hadiya zone, Southern Ethiopia: Unmatched case-control study
Source: PLoS One. 2021 May 12;16(5):e0250548. doi: 10.1371/journal.pone.0250548 (PMC8115896; doi:10.1371/journal.pone.0250548)
Supplement: S2 File — (DOCX) [file pone.0250548.s002.docx]

**Annex I**: **English version participants information sheet and consent form**

Title: ***Determinants of pregnancy induced hypertension on maternal and foetal outcomes in Hossana town administration, Hadiya zone, Southern Ethiopia: Unmatched case-control stud.***

Address: Phone (mob.) 0923406980/0913791113

Email: [gossabo2004@gmail.com](mailto:gossabo2004@gmail.com)

Name of the organization Wachemo University school of medicine & health science, department of nursing

**Information to study participants**

Greeting --------------------------------------------------------

My name is------------------------- I am instructor in wachemo University. I am planning to conduct my study on maternal, perinatal outcomes of pregnancy indiced hypertension and associated factor among pregnant mothers at Hossana town, in Hadiya Zone, Southern Ethiopia. I am going to ask you some questions that are not difficult to answer. Whatever information you provide will be kept confidentiality. Your name will not be written in this format, never be used in connection with any of the information you are going to tell me and your name is not identified in any of this output. You are not obliged to answer any question that you do not want to answer and you may drop-out this interview at any time you want to. However, your honest answers to these questions will help us in identifying the determinant factors of pregnancy induced hypertension in the future.

We would appreciate your help in responding to these questions and we value your input to make this study a successful one

**Purpose of the study**

The ultimate purpose of this study is to determine maternal, perinatal out pregnancy induced hypertension outcomes among pregnant women at Hossana town. Thus, the information that you will provide us help to assess the determinants of pregnancy induced hypertension and improves service utilization and strategy to manage outcomes. In summary, the study will assess how many and why came with negative impact of the pregnancy induced hypertension. Results from this study will be used to assist in making recommendations for those who are responsible to design effective and appropriate measure to increase early screening and management of the pregnancy induced hypertension.

**Conformation of Consent to participate**

Do you understand all I have just told you and do you agree to participate in this study? If you agree to participate in this study, you will need to give consent.

Yes ----------------------continue No------------------------stop

**PARTICIPANT AGREEMENT:- Participant**: I have read the study information and understand its objective / have been briefly informed about the study that has been read to me. I have been asked if I have any questions, and these have been answered to my satisfaction. I freely agree to participate.

Agree disagree

**Data collector**: I certify that the nature and purpose, the potential benefits, and possible risks associated with participating in this research have been explained to the above individual, and the individual has consented/agreed to participate.

Name Data collector __________________signature _________________date _________

| Code | Variable | | | Category /response | Skip |
| --- | --- | --- | --- | --- | --- |
|  | **Part I: Socio-demographic variables** | | | |  |
| 101 | Age of women during the current pregnancy | | | ------- |  |
| 102 | Educational status | | | 1. unable to write & read 2. able to read and write 3. 1-8^th^ 4. 9^th^ – 12^th^ 5. College & above |  |
| 103 | Occupation | | | 1. Household wife 2. Gov’t employee 3. NGO employee 4. Self-business 5. Student |  |
| 104 | Marital status | | | 1. single 2. married 3. divorced 4. living together |  |
| 105 | Religion | | | 1. orthodox 2. protestant 3. Muslim 4. Seventh day advents 5. Others |  |
|  | **Part II: Obstetric d and gynaecological history** | | | |  |
| 201 | Gravidity | | | 1. Primgravida 2. II 3. III & above |  |
| 202 | Parity | | | 1. 0 2. 1-2 3. 3-4 4. More than 5 |  |
| 203 | Do you remember your LNMP of current conception? | | | 1. Yes 2. No | Skip to Q 205 |
| 204 | If your answer for Q203 is yes, what is GA in weeks ( on the occasion of current visit) | | | ---- |  |
| 205 | Women Gestational age confirmed by US (if) | | | ---- |  |
| 206 | Did you attend ANC follow up? | | | 1. Yes 2. No | Skip to Q 208 |
| 207 | If your answer for Q 206 is yes, How many ANC follow-up do you have | | | 1. I 2. II 3. III 4. IV and above |  |
| 208 | Pregnancy (confirmed by health professionals) | | | 1. Single 2. Multiple |  |
| 209 | Do you have any previously diagnosed chronic disease (by health professionals) | | | 1. Yes 2. No | Skip to Q 211 |
| 210 | If your answer for Q 208 is yes Which diagnosed medical disorder do you have  ( More than one answer is possible) | | | 1. DM 2. Anemia 3. Non PIH 4. Heart disease 5. Seizure disorder 6. Others specify----- |  |
| 211 | Previous history of pregnancy Induced hypertension | | | 1. Yes 2. No |  |
|  | **Part III: Mothers status on admission** | | | |  |
| 301 | Chief complaint during admission  (More than one answer is possible) | | | 1. Headache 2. Blurred vision 3. Epigastric pain 4. Nausea and vomiting 5. Convulsion 6. Edema (pedal) 7. Dizziness |  |
| 302 | Blood pressure during admission | | | DBP ------  SBP ------ |  |
| 303 | Condition of patient presentation on arrival to Hospital | | | 1. Conscious 2. Semi -conscious 3. comatose 4. convulsion |  |
| 304 | Does mother developed PIH (based on the assessment and documents ) | | | 1. yes 2. No |  |
| 305 | Category of hypertension during admission | | | 1. chronic hypertension 2. preeclampsia 3. Eclampsia 4. Peeclampsia superimposed on chronic HTN |  |
|  | **Part IV: Investigation done** | | | |  |
| 401 | Does blood group & Rh done | | 1. Yes 2. No | |  |
| 402 | Protein urea | | 1. Null 2. +1 3. +2 4. +3 5. > +4 6. Note done | |  |
| 403 | Platelet count | | 1. <100,000 2. 100,000 – 150,000 3. >150,000 4. Not done | |  |
| 404 | Does LFT done | | 1. Yes 2. No | | Skip to Q 406 |
| 405 | LFT Result | | AST------  ALT ----- | |  |
| 406 | Does RFT done | | 1. Yes 2. No | | Skip to Q 501 |
| 407 | RFT Result | | Creatinen ------  BUN ------- | |  |
|  | **Part V: onset of labor and delivery** | | | |  |
| 501 | Which type of onset of labor she had | 1. Spontaneous 2. Induction | | |  |
| 502 | Mode of the delivery | 1. Spontaneous vaginal delivery/SVD 2. Cesareans section/SC | | |  |
| 503 | If answer for Q 502 is 2, what was an indication for it | 1. Uncontrolled peeclampsia 2. Happening of Eclampsia 3. Failure to Induction or augmentation 4. Abroptio placenta 5. Fetal distress 6. Others specify | | |  |
|  | **Part VI: maternal and foetal outcomes** | | | |  |
| 601 | Is there any maternal complication | 1. Yes 2. No | | |  |
| 602 | If answer for Q 601 is yes, Which one | 1. HELLP Syndrome 2. Eclampsia 3. DIC 4. Cardiac failure 5. Renal failure 6. PPH 7. Pulmonary edema 8. Others specify | | |  |
| 603 | Maternal death | 1. Yes 2. No | | |  |
| 604 | Fetal outcome of delivery | 1. A live birth 2. Alive but admitted in NICU 3. Still birth | | |  |
| 605 ‘A’ | IF life birth, what was APGAR score for Neonate A | 1. Within 1^st^ minute ----- 2. At 5 minute ----- | | |  |
| 605 ‘B’ | IF life birth, what was APGAR score neonate B | 1. Within 1^st^ minute ----   2 At 5 minute --- | | |  |
| 606 ‘ A’ | What was fetal weight per Kg for fetus A | 1. < 1.5 2. 1.5- 2.5 3. 2.5-4.0 4. > 4.0 | | |  |
| 606 ‘B’ | What was fetal weight per Kg fetus A | 1. < 1.5 2. 1.5- 2.5 3. 2.5-4.0 4. 4.0 | | |  |
| 607 | Gestational age at delivery | 1. Pre-Term 2. Term 3. Post term | | |  |
| 608 | Neonatal death | 1. Yes 2. No | | |  |
| 609 | Does a neonate had IUGR | 1. yes 2. No | | |  |

**Thank you!**
