# Supplementary material for: Determinants of pregnancy-induced hypertension on maternal and foetal outcomes in Hossana town administration, Hadiya zone, Southern Ethiopia: Unmatched case-control study
Source: PLoS One. 2021 May 12;16(5):e0250548. doi: 10.1371/journal.pone.0250548 (PMC8115896; doi:10.1371/journal.pone.0250548)
Supplement: Attachment — Submitted filename: Response to reviewer .docx [file pone.0250548.s003.docx]

**Response to reviewers**

All authors of this manuscript read and took any effort to revise the whole manuscript based on the comment given by peer review. For all forwarded minor and major comments authors gave response as follows.

1. We assure you that all components (manuscript, tables, figures, abstract and abbreviations) of this article was prepared based on the PLOS ONE guide line.
2. My ORCID is 0000-0002-7214-4025
3. Abstract in the manuscript and online submission in-terms of character and contents were revised and identical.
4. For all listed figure (figure_1 & Figure_2) in the main part of the manuscript their copies uploaded separately.
5. Copy of tables (table 1-5), which referred in the text were uploaded immediately next to the each paragraph in the manuscript.

Reviewer #1: The finding of this study revealed that women with previous history of pregnancy induced hypertension more likely to develop pregnancy induced hypertension on current conception whereas women with ≥ 3 number of pregnancies and informal education status were decreased odds of pregnancy induced hypertension. Thus, research team/authors recommend that concern bodies should have facilitate basic setups to make conducive environment (women waiting village, in-service training, and tax free transportation) for better management of the cases where as health care providers should have increased early diagnosis and on time intervention rather than managing complication. Therefore, our paper conclusion part is modified as like aforementioned.

**Major comment**

1. Conclusion in abstract and paper regarding factors revised based on the finding of the multivariate logistic regression. Variables description: women with previous history of pregnancy induced hypertension increased odds of developing PIH in current conception whereas number of pregnancies ≥ 3 and women having informal education status are 67.7%, 68.4% less likely to develop pregnancy induced hypertension/PIH respectively, this implies they associated with reduced odds of developing PIH. From the point of epidemiology, if the odd ratio/OR < 1 a factor has protective effect of likelihood occurrence of event. Therefore, both number of pregnancies ≥ 3 and women having informal education status reduced odds of developing pregnancy induced hypertension/PIH.

2. In fact, background of this manuscript contains detail about others studies, but for seek of readers better understanding and readability background of the manuscript was revised based on the stated objectives and its finding. As you have reviewed the back ground of the manuscript contained detail about the previously studied articles, but the revised background contain about Pregnancy induced hypertension of preeclampsia and eclampsia and maternal and foetal outcomes in details. Moreover, discussion part also restructured through focusing on Objectives (predictors of PIH and foeto-maternal outcomes) and for more understanding almost all comparison was made with case control studies. We assure you that all comparison made based on the reference which in-line with our study. For further accuracy and consistent you can crosscheck according to the cited reference.

**Minor comment**

3. The manuscript for better readability and understanding edited by English language professionals who is PhD candidate in Addis Ababa University, Ethiopia. If you have further enquires you can communicate through this address. Email [negatuhabitamu@gmail.com](mailto:negatuhabitamu@gmail.com) cell phone +251912191000.
4. Pregnancy induced hypertension and preeclampsia differ according to the ‘operational definition given in this manuscript. In-addition, Pregnancy induced hypertension is a broad definition and according to the latest American college of obstetrics and gynaecologist preeclampsia is one category of Pregnancy Induced hypertension/PIH. In-fact, Preeclampsia may lead to ecalmpsia but not Pregnancy induced hypertension lead to preeclampsia. A woman diagnosed with preeclampsia who experienced with convulsion with or without coma can be diagnosed as eclampsia.
5. Words ‘likelihood’, ‘prone’ used to describe finding of the multivariate analysis are revised according the given comment. In main manuscript multivariable analysis finding (Odds ratio) revised as previous history of pregnancy induced hypertension is increases odds of developing PIH in current conception whereas informal educational status decrease odds of PIH or less likely develop pregnancy induced hypertension.
6. For all reported percentages in the text cross-ponding ‘numbers’ also added. For further confirmation you can check highlighted text under revised manuscript.
7. Standard Abbreviation appears first in text fully described and appears in text less than three times were excluded.
8. As our understanding if we combine any one of the table; readers may be confused because their descriptions and contents are different. For those abbreviations in the table which are not described under abbreviation explanations in text are given the tables.
9. All structured and pretested questionnaires are uploaded as supporting document.
