# Supplementary material for: Determinants of pregnancy-induced hypertension on maternal and foetal outcomes in Hossana town administration, Hadiya zone, Southern Ethiopia: Unmatched case-control study
Source: PLoS One. 2021 May 12;16(5):e0250548. doi: 10.1371/journal.pone.0250548 (PMC8115896; doi:10.1371/journal.pone.0250548)
Supplement: Attachment — Submitted filename: Response to reviewres.doc [file pone.0250548.s004.doc]

**Response to reviewers**

All authors of this manuscript read and took any effort to revise the whole manuscript based on the comment given by the reviewer. All authors try to incorporate the given comment in the entire manuscript. For all forwarded minor and major comments authors gave response as follows.

All authors rigorously tried to address all points answered by reviewer, which stated from 1-7. Then major improvements were made from abstract to recommendation.

Reviewer #1:

Major

In addition to previous edition made by English language professional who is PhD candidate in Addis Ababa University, Ethiopia, in this round additional edition to improve readability and understanding made by Dr. Feleke Doyore (PhD, health promotion and communication) Who is lecturer in Wachemo University) and English language professional Mr Yirga H. (BA, MA) currently, who is teaching in Bobicho preparatory school, Hossan, Ethiopia.

Minor

Regarding Pregnancy Induced Hypertension, Pre-eclampis and eclampsia; to improve readability and understanding for the reader, detail definition written in the background part (from line number 85-94). In addition to this, for further understanding detail definition is also described in the operational definition part (line number 324-332).

The relation between pregnancy induced hypertension with preeclampsia and eclampsia is one is the inclusive the other; when a women diagnosed for eclampsia also fulfil diagnosis criteria of preeclampsia as well women diagnosed for preeclampsia fulfil diagnosis criteria of pregnancy induced hypertension. Therefore, eclampsis = definition of Preeclampsia + occurrence of convulsion or coma + ≥^+^2 proteinuria whereas preeclampsia = definition of pregnancy Induced hypertension + presence of proteinuria in dipstick test with or without oedema.

The effect of the pre-eclampsia and eclampsia on the maternal and foetal health explained in detail in the background part (from line number 132-153), which reflect the related outcomes of preeclampsia and eclampsia.

Sample size calculation was performed by using statistical software EPI-Info V 7 for window considering all statistical assumptions and others those needed to estimate sample size. Thus, software sample size estimation is more recommendable and it gives large sample size than manual calculation.

All figures of the ORs, CIs and P values in the tables 4 and 5 are changed to the 2 decimal places from 3 decimal places.

Table 5 finding and text report regarding having ≥3 previous pregnancies and its odds ratio edited as ‘’ decrease odds of pregnancy induced hypertension.’’

**Discussion**

Discussion based on the objective of the study revised rigorously. It is the fact that, the first objective of this study is to measure the outcomes of the pregnancy induced hypertension among cases and controls group. Hence, the outcomes of pregnancy induced hypertension on maternal and foetal health explained in detail and based on the multivariate analysis predictors of pregnancy induced hypertension discussed one by one.

Conclusion

To improve understanding and readability for the readers, major amendment undertaken in conclusion part based on the objective of the study and finding of multivariate analysis.
