# Supplementary material for: Determinants of pregnancy-induced hypertension on maternal and foetal outcomes in Hossana town administration, Hadiya zone, Southern Ethiopia: Unmatched case-control study
Source: PLoS One. 2021 May 12;16(5):e0250548. doi: 10.1371/journal.pone.0250548 (PMC8115896; doi:10.1371/journal.pone.0250548)
Supplement: Attachment — Submitted filename: Response to reviewres.doc [file pone.0250548.s005.doc]

**Response to reviewers**

Under the guidance of corresponding authors, all authors read for long period and they brought the revised manuscript to the table and revision was made together. The whole manuscript revised based on the comment given by the reviewer though using copyedit and others language academicians. For all forwarded major and minor comments authors gave response as follows.

All authors rigorously tried to address all points answered by reviewer, which stated from 1-6. Then major improvements were made from abstract to recommendation according the points raised by reviewers.

Reviewer #1:

In addition to previous edition made by English language professional who is instructor at Bobicho high school, Hossana, Ethiopia, Mr Yirga H. (BA, MA) in this round additional edition in every section (abstract to Conclusion) to improve readability and understanding made by Amanuel Tirkaso (MA, PhD candidate) Who is lecturer at English department, college of social science, Wachemo University, Hossan

In order to avoid unnecessary repetition, exhaustive reading and formatting few lines were undertaken. To minimize paper length, total words in main body of the manuscript (introduction to conclusion) reduced from 5674 to 5,222 words.

Regarding Pregnancy Induced Hypertension, Pre-eclampis and eclampsia definition and relation; to improve readability and understanding for the reader, detail definition written in the background part (from line number 83-89). In addition to this, for further understanding detail definition is also described in the operational definition part (line number 289--298).

The relation between pregnancy induced hypertension with preeclampsia and eclampsia is one is the inclusive the other; a woman diagnosed for pre-eclampsia characterised with elevated blood pressure (SBP ≥ 140 & DBP ≥ 90mmHg) without presence of proteinuria which developed after 20 weeks of gestation. Preeclampsia: two reading of SBP ≥ 140 mmHg & DBP ≥ 90mmHg 4-6 hours apart after twenty weeks of gestation with proteinuria 2+ or more whereas eclampsia is women with signs and symptoms of preeclampsia plus convulsion with or without coma. Oligohdrouia or anuria is present. Therefore, eclampsis = definition of preeclampsia + occurrence of convulsion or coma + ≥^+^2 proteinuria whereas preeclampsia = definition of pregnancy-induced hypertension + presence of proteinuria in dipstick test with or without oedema.

All miss spelt words edited appropriately. Abbreviation correction in case of first appearance revised through out of the main body of the manuscript. Regarding Intra uterine growth retardation/IUGR the right word is retardation but not restriction.

**Discussion**

Discussion based on the stated objective of the study revised rigorously. More focuses of the discussion done considering studies done using case control design. Hence, the outcomes of pregnancy induced hypertension on maternal and foetal health explained in detail and based on the multivariate analysis predictors of pregnancy induced hypertension discussed one by one.

Conclusion

Based on the objective of the study, conclusion focus adverse outcomes of PIH on maternal and foetal health and multivariate analysis findings.
